# Development and validation of teacher and student questionnaires measuring inhibitors of curriculum viability

**DOI:** 10.1186/s12909-021-02843-0

**Published:** 2021-07-28

**Authors:** Rehan Ahmed Khan, Annemarie Spruijt, Usman Mahboob, Mohamed Al Eraky, Jeroen J. G. van Merrienboer

**Affiliations:** 1 Islamic International Medical College, Riphah International University, Al-Mizan IIMCT Complex, Old Supreme Court Building, 274 Peshawar Rd, Rawalpindi, Pakistan; 2 School of Health Professions Education, Maastricht University, Maastricht, the Netherlands; 3 Faculty of Veterinary Medicine, Utrecht University, Utrecht, the Netherlands; 4 Institute of Health Professions Education and Research, Khyber Medical University, Peshawar, Pakistan; 5 Imam Abdulrahman Bin Faisal University, Dammam, Saudi Arabia

**Keywords:** Curriculum, Standards, Evaluation, Viability inhibitors, Construct validity

## Abstract

**Background:**

Curriculum viability is determined by the degree to which quality standards have or have not been met, and by the inhibitors that affect attainment of those standards. The literature reports many ways to evaluate whether a curriculum reaches its quality standards, but less attention is paid to the identification of viability inhibitors in different areas of the curriculum that hamper the attainment of quality. The purpose of this study is to develop and establish the reliability and validity of questionnaires that measure the presence of inhibitors in an undergraduate medical curriculum.

**Methods:**

Teacher and student questionnaires developed by the authors were sent to medical educationalists for qualitative expert validation and to establish their content validity. To establish the response process validity, cognitive interviews were held with teachers and students to clarify any confusion about the meaning of items in the questionnaires. Reliability and construct validity of the questionnaires were established by responses from 575 teachers and 247 final-year medical students.

**Results:**

Qualitative expert validation was provided by 21 experts. The initial teacher and student questionnaires containing respectively 62 items to measure 12 theoretical constructs, and 28 items to measure 7 constructs, were modified to improve their clarity and relevance. The overall scale validity index for the questionnaires was, in order, .95 and .94. Following the cognitive interviews, the resultant teacher and student questionnaires were reduced to respectively 52 and 23 items. Furthermore, after the confirmatory analysis, the final version of the teacher questionnaire was reduced to 25 items to measure 6 constructs and the student questionnaire was reduced to 14 items to measure 3 constructs. Good-for-fit indices were established for the final model and Cronbach alphas of, in order, .89 and .81 were found for the teacher and student questionnaire.

**Conclusion:**

The valid and reliable curriculum viability inhibitor questionnaires for teachers and students developed in this study can be used by medical schools to identify inhibitors to achieve standards in different areas of the curriculum.

**Supplementary Information:**

The online version contains supplementary material available at 10.1186/s12909-021-02843-0.

## Background

Curriculum quality is typically assessed through curriculum evaluation [1], which determines the quality of a curriculum by assessing its various aspects against a particular set of standards. This process, however, does not explicitly involve finding the issues that inhibit meeting specific standards. The issues impeding the achievement of curriculum quality standards are called ‘curriculum viability inhibitors’ [2]. Together, the presence of current inhibitors in the curriculum and the degree to which relevant standards are met make up the ‘viability indicators’, which determine the curriculum viability [3]. Many questionnaires reportedly measure attainment of quality standards in different areas of the curriculum. For instance, DREEM, AMEET, HELES [3–5] and JHLES [6] measure the educational environment, and AIM measures the implementation of assessment [7]. Yet we did not find any questionnaires that measure the inhibitors of the curriculum. Knowledge of inhibitors is particularly useful for reviewers when an existing curriculum needs to be renewed. Curriculum developers can also consider the inhibitors during the process of curriculum development, taking preventive measures to design a curriculum that has minimal issues when implemented.

Inhibitors of curriculum quality can also be explored through interviewing the stakeholders about different aspects of curriculum. However, that requires ample time and data analysis and involves perception of a rather small number of respondents compared to survey questionnaires. Certain tools developed by accreditation bodies use open-ended qualitative questionnaires to solicit views of medical educationalists or members of medical education departments [8]. Although medical educationalists are curriculum experts in a general sense, they may not be expert in viability inhibitors of a specific curriculum perceived and practiced by medical students and teachers at large. Therefore, there is a need to develop questionnaires that can easily be interpreted by all stakeholders involved in identifying inhibitors. The aim of this study is therefore to develop and establish the validity and reliability of student and teacher questionnaires measuring viability inhibitors.

In an earlier study, a scoping review on curriculum viability indicators showed 37 standards and 19 inhibitors [2]. Thirteen studies dealt with standards, but only two studies described both standards and inhibitors. Thus, a Delphi study was conducted to develop consensus on curriculum viability inhibitors among experts [3].

The main stakeholders of the curriculum in a medical college are teachers, students, and educational managers. Though educational managers have a significant stake in the implementation and development of the curriculum, the curriculum is mainly implemented by the teachers and experienced by the students. Accordingly, this study addresses the following questions covering the steps of development and validation of a questionnaire [9]: (1) What items in a teacher and student questionnaire are relevant to measure curriculum viability inhibitors according to medical education experts (*Expert validation*)? (2) What is the *content validity* of the teacher and student questionnaires? (3) How do teachers and students interpret the items in the teacher and student questionnaire *(Response Process Validity)*? And (4) what are the *construct validity* and *reliability* of the questionnaires?

## Methods

### Study design and settings

**Fig. 1:**
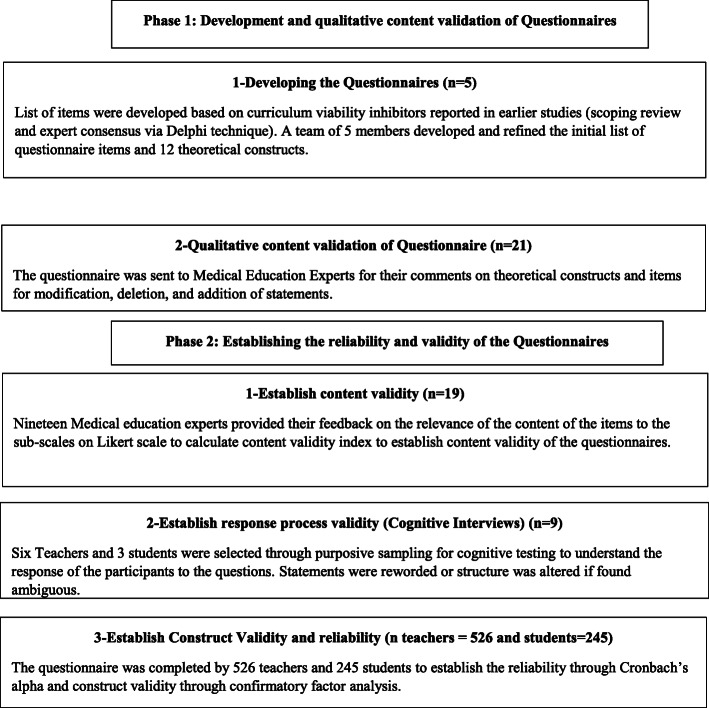
Phases of the study. Phase 1 and 2 of the study that show development and validation of the teacher and student questionnaires measuring curriculum viability inhibitors

Development and validation of the curriculum viability inhibitor questionnaires comprised two main phases, as shown in Fig. 1. The first phase was the development of questionnaires and getting qualitative expert feedback to refine them. The second phase was establishing the content validity, response process validity, construct validity, and reliability of the questionnaires.

Defining and measuring the inhibitors that constitute the theoretical constructs in the questionnaires will help an educational institution find the issues that hamper the attainment of a healthy curriculum and hence to develop ‘treatments’ for improving curriculum viability. Some of these theoretical constructs include irrelevant curriculum content, low quality assessment, lack of social interaction, and lack of sharing best practices. Table 1 shows all the 12 theoretical constructs with their descriptions.

**Table 1:** Inhibitors and their definitions

Inhibitor	Definition
**Irrelevant curriculum content**	Curricular content that does not match with curricular outcomes.
**Lack of resources in an institution**	Resources that are not available according to the requirement of the course/ curriculum such as funds for library, ICT facilities, support staff, student advisors etc.
**Low quality assessment**	Assessment that is not aligned to instructional methods and content distribution and is not in accordance with principles of assessment.
**Lack of sufficient time for studying**	Less time available to students for self-study and exams.
**Neglecting student needs and requirements**	Students’ influence on the curriculum such as student evaluations and feedback taken into account when the curriculum is renewed or when new courses are developed.
**Presence of strong disciplinary cultures**	Culture over-concerned with procedures at the expense of efficiency, having more focus on inspection and control.
**Lack of social interaction**	Lack of interaction between the faculty and students and among them.
**Research culture and patient care undervaluing education**	Research or patient care is given more importance than teaching in terms of promotion and funding.
**Lack of policies and procedures**	Lack of formal policies and procedure documents in the institution affecting the curriculum and their implementation.
**Leaders acting as communication gatekeepers**	Leaders withholding, delaying, or passing selected information to all relevant stakeholders, e.g., teacher, students, educational managers, and maybe even others.
**Lack of staff involvement in organizational decision-making**	Lack of staff involvement in decisions that affect the curriculum, e.g., on the content of courses, time schedules, kind of educational activities, use of ICT etc.
**Lack of sharing best practices across organisation**	Lack of sharing existing practices that already possess a high level of widely agreed effectiveness.

This study was approved by the Institutional Review Committee at Riphah International University (Appl. # Riphah/IRC/18/0394). Written informed consent was taken from all the participants.

The study duration was from October 2019 to July 2020. It was conducted involving medical education experts, students, and teachers from various institutions; the details of which have been provided in phase 1 and 2 in the relevant sections.

### Phase 1

In this phase, answering our first question, the authors developed the first version of the teacher and student questionnaires based on literature review, and refined the questionnaires after receiving qualitative feedback from expert medical educationalists.

#### Development and qualitative content validation of teacher and student questionnaires

##### Participants, materials and procedure

Out of 27 experts who were invited based on their qualifications (at least Master’s in medical education or equivalent qualification) and experience in medical education (more than 5 years), 21 (77%) responded and provided feedback on the first version of the questionnaire, with comments on the constructs and related items.

The first version of the teacher questionnaire had 62 items measuring 12 constructs, whereas the student questionnaire had 28 items measuring 7 constructs.

The first author (RAK) developed the items for measuring each inhibitor based on a scoping review [2] and a consensus-building Delphi study amongst a group of experts [3]. The co-authors (AS, UM, MAE, and JJM) then refined the questionnaire before sharing it with medical education experts through e-mail. The experts were asked to provide qualitative feedback on the questionnaire items to improve their clarity and relevance to the inhibitor if needed, and also to comment on deletion or addition of items.

##### Data analysis

The feedback was initially analysed by the first author by organizing the comments on the items. The changes in the items suggested by experts were made based on the criteria: (1) item easy to understand, (2) relevant to the construct, (3) avoid duplication or similar meanings, (4) minimize grammatical and formatting errors, and (5) avoid double-barreled statements. The questionnaire was then shared with co-authors for their feedback and consensus on modifications to the items.

Based on the expert feedback, items were reworded for clarity and grammatical inaccuracies or deleted if found not relevant to the construct or having a meaning very similar to another item. Some items were shifted to another construct if they were not found suitable for their current construct. When multiple suggestions were given for a single item, the commonly suggested modification was used and was finalized by the discussion and agreement of the authors.

### Phase 2

This phase comprised of three steps: (1) establishing the content validity, (2) response process validity, (3) construct validity, and reliability of the questionnaires.

#### Step 1: establishing the content validity of teacher and student questionnaires

##### Participants, materials and procedure

To rank the items for content relevance and clarity, 19 out of 21 (90.5%) medical education experts from Phase 1 participated in Phase 2*.*

The revised questionnaire (version 2) based on the feedback from the medical education experts; for teachers had 60 items measuring 12 constructs (see Additional file 1: Appendix A), for students, it had 28 items measuring 7 constructs (see Additional file 1: Appendix B). For both questionnaires, Likert scales were used to measure the relevance and clarity of the items. For relevance we used: 4 = very relevant, 3 = quite relevant, 2 = somewhat relevant, and 1 = not relevant. For clarity, we used: 3 = very clear, 2 = item needs revision, and 1 = not at all clear.

The questionnaire version 2 was sent via email to 21 experts who had previously provided feedback in Phase 1, with a request to respond within 3 weeks. They were asked to score the items on the Likert scales and provide feedback to improve the items further. Out of 21 participants, 19 responded. The forms sent by 5 participants were incomplete and they were requested to send the completed forms. Only two participants complied, hence a total of 16 complete forms were included in the study.

##### Data analysis

To establish content validity, quantitative and qualitative data were analysed. For the quantitative component, the content validity index (CVI) for the individual items (I-CVI), and of the scale (S-CVI) were calculated [9], based on the scores given by the experts.

I-CVI was calculated as the number of experts in agreement divided by the total number of experts, and S-CVI was determined by calculating the average of all CVI scores across all the items. To calculate I-CVI, the relevance ratings of 3 or 4 were recoded 1, and items ranked 1 or 2 were recoded as 0. For each item, the 1 s were added and divided by the total number of experts to calculate the I-CVI.

To improve the clarity of the items where a 3-point Likert scale was used, the content clarity average was calculated. The average clarity of an individual item was calculated by adding the sum of all the values given to the item divided by the total number of items. Average clarity above 2.4 (80%) was considered to be very clear [10].

The comments provided by the experts were categorized into general comments for the questionnaire and specific comments for the items. Based on these comments, the items were modified.

#### Step 2: establishing response process validity through cognitive interviews

Cognitive interviewing is a technique that validates the understanding of items in a questionnaire by the respondents.

##### Participants, materials and procedure

Interviews were held with 6 teachers, 3 each from basic and clinical faculty to have representation from basic and clinical sciences, and 3 students from the final year MBBS as they have the maximum exposure to the curriculum .

In version 3, the teacher questionnaire had 53 items measuring 12 constructs, and the student questionnaire had 23 items measuring 7 constructs. We used a combination of ‘think aloud’ and ‘verbal probing’ techniques [9].The participants were asked to read the item silently and think aloud what came to their mind after reading it [11]. In verbal probing, we asked scripted and spontaneous questions after the participant had read an item [12]. We combined the verbal probing and think-aloud techniques, as ‘think aloud’ acts as a cue for respondents, to yield additional information on the quality of the items as explained in the procedure section below.

Test interviews were conducted with 1 co-author, 1 teacher, and 1 student using Zoom (zoom.us) to identify possible issues related to combining think-alouds and verbal probing. The time participants needed to answer the items in the questionnaire was also determined. The average cognitive interview lasted approximately 60 min for 27 items in the teacher questionnaire and 50 min for 23 items in the student questionnaire. We also piloted cued retrospective probing [13], in which the primary researcher replayed the recorded think-aloud to the participant and explored the items with scripted and spontaneous probes. We found that it yielded no extra benefit in providing a cue as compared to the combination technique and also required more time.

The protocols regarding cognitive interviews for the study were planned based on the pilot interviews as they require a sustained concentration on behalf of the participants [14]. Hence for the teacher questionnaire, we divided the 53 items in the questionnaire between 2 participants whereas the student questionnaire did not require division as it had only 23 items. To increase the credibility of the interview technique and reduce bias, another researcher (UM) was also present during each interview.

##### Data analysis

Analytic memos were created based on the think-aloud and verbal probing. These memos were coded into the following categories: (1) items with no problems in understanding, (2) items with minor problems in understanding, and (3) items with major problems in understanding [15]. These categories were assigned independently by RAK and UM. Items that required more clarity were reworded and further refined through review from the remaining co-authors (AS, MAL, and JVM). The details of the response process validity for the purpose of reproducibility are provided in the Additional file 1: Appendix C.

#### Step 3: establishing reliability and construct validity

##### Participants, materials and procedure

Based on the adequate sample size (minimum of 10 participants per item) reported in the literature, our target sample was 520 teachers for 52 items and 230 final-year medical students for 23 items [16, 17] in the respective questionnaires. A total of 575 teachers from 77 medical colleges and 247 final-year students from 12 medical colleges filled out the questionnaire. We selected those teachers who were currently involved in teaching and had been involved in implementing or developing the curriculum. Curriculum involvement was described as the development of module or course and teaching, assessing, and managing it. Final-year medical students were recruited, as they have the maximum experience of the curriculum. The designation, academic qualification, experience of teaching, experience in medical education, and type of curriculum practiced is shown in Table 2. Out of the 575 teachers, 526 provided complete responses, whereas 245 out of 247 students provided complete responses.

**Table 2:** Participant Demographics for confirmatory factor analysis of teacher questionnaire (*N* = 526)

Designation	Qualification in Medical Education	Experience as a Teacher	Experience in Medical Education	Type of Curricula Practiced in the Institution
Professor (22%)	PhD (3%)	> 20 years (7%)	> 20 years (2%)	Discipline-based (29%)
Associate Professor (18%)	Master’s (44%)	16–20 years (10%)	16–20 years (1%)	Integrated (35%)
Assistant Professor (30%)	Fellowship (22%)	11–15 years (21%)	11–15 years (7%)	Problem-based (4%)
Senior lecturer (13%)	Diploma (4%)	5–10 years (30%)	5–10 years (18%)	Theme-based (3%)
Lecturer (17%)	Certificate (17%)	< 5 years (32%)	< 5 years (72%)	Hybrid (Mix of Discipline and Integration) (29%)
	Workshops only (10%)			

The fourth version of the teacher questionnaire had 52 items measuring 12 constructs, and the student questionnaire had 23 items measuring 7 constructs. The items had to be scored on a 5-point Likert scale: 1 = strongly disagree, 2 = somewhat disagree, 3 = neither agree nor disagree, 4 = somewhat agree, and 5 = strongly agree. The items were shuffled so that they were not grouped by the hypothesized constructs. We also shuffled the answer options in a few items and informed the respondents. We did this so that questions were carefully read and answered by the respondents to encourage response *optimizing and prevent satisficing* [18–20].

A pilot study of the questionnaire was conducted with 20 teachers and 15 medical students to ensure the smooth working of the Qualtrics link (www.qualtrics.com) and resolve any difficulty browsing through the questionnaire. No issues were reported by the participants. To maximize the response, we shared the questionnaire link through different sources. The link was sent to the Deans and Directors of medical education of the colleges through emails. They were also shared with the master’s in health professions students in their WhatsApp Groups. ﻿The invitation message stressed the formative purpose and use of the evaluations and the confidential and voluntary character of participation. To encourage participation, e-mail reminders were sent on Day-5 and Day-10, apart from reminders through WhatsApp to the Directors of medical education departments.

##### Data analysis

To ascertain the internal structure of the questionnaire, internal consistency was calculated through Cronbach’s Alpha. Then, we conducted confirmatory factor analysis (CFA) as we had specific expectations regarding (a) the number of factors (constructs/subscales), (b) which variables (items) reflect given factors, and (c) whether the factors correlated [21].

The questionnaires were evaluated using SPSS version 26 and AMOS version 26. Regarding internal consistency, Cronbach’s alpha of between .50 to .70 was considered a satisfactory internal consistency for the scale and subscales [22–24]. Corrected item correlation test (CITC) was calculated for the items of the subscales that had low internal consistency. CITC in the range of .2 to .4 was considered an acceptable value to retain the item [25, 26].

Construct validity was established via CFA. For the goodness-of-fit of the measurement model, we measured the absolute, incremental, and parsimonious fit indices. Absolute fit indices assess the overall theoretical model against the observed data, incremental or comparative fit indices compare the hypothesised model with the baseline or minimal model, whereas the parsimonious fit model index assesses the complexity of the model [27, 28]. The indices used for absolute fit are root mean square error of approximation (RMSEA) < .05 as a close fit, < .08 as an acceptable fit [29], and goodness-of-fit index (GFI) > .90 as a good fit [30]. For incremental fit, the indices considered acceptable are comparative fit index (CFI) > .90, adjusted goodness of fit index (AGFI) > .90, Tucker Lewis Index (TLI) > .90 [31], and normed fit index > .90 [32]. For parsimonious fit, Chi-square difference (χ^2^/df) < 5.0 is considered acceptable [4, 33].

## Results

### Phase 1: development of the questionnaires

Based on the feedback provided by experts on the first version of the teacher’s questionnaire, 5 of 62 items were deleted as they were being duplicated; 43 items were modified because they required rewording for clarity based on incorrect grammar, formatting errors, and understandability; and 3 new items were added. The result was the next version having 60 items, as shown in Table 3.

**Table 3:** Modifications done in different versions of the teacher and student questionnaires

	Expert Feedback	Content validity	Response Process Validity	Construct Validity	
	Questionnaire Version 1	Questionnaire Version 2	Questionnaire Version 3	Questionnaire Version 4	Questionnaire Version 5 (final)
**Teacher questionnaire**					
Total Items	62	60	53	52	25
Items accepted without change	14	16	42	–	–
Items accepted after modification	43	36	10	–	–
Items deleted	5	8	1	27	–
New Items added	3	1	–	–	–
Final items	60	53	52	25	–
**Student questionnaire**					
Total Items	28	28	23	23	14
Items accepted without change	6	6	16	–	–
Items accepted after modification	22	17	7	–	–
Items Deleted	–	5	–	9	–
Final items	28	23	23	14	–

Regarding the student’s questionnaire, 22 of 28 items were modified while 6 items were not changed. Among the 22 items modified, 21 items were reworded for lack of clarity and grammatical inaccuracies (Table 3).

### Phase 2: establishing the validity and reliability of the questionnaires

#### Content validity index and content clarity average of the teacher’s questionnaire

Out of 60 items, 4  items had a CVI less than .70 and were removed, 3 items had a CVI between .70 and .79; they were modified according to the qualitative feedback of the experts and retained. The remaining items had a CVI higher than .79. However, the experts indicated that 4 items were similar in meaning to other items and were therefore also removed. The third version of the questionnaire thus had 53 items. Overall scale content validity (SCVI/AVG) of the questionnaire was .95.

Out of 53 items, 7 had a content clarity average (CCA) of 3 (100% clarity), 38 between 2.75 and 2.93, and 12 between 2.56 and 2.68. The average clarity of the scale was 2.81. Based on the qualitative feedback, 36 items in the questionnaire were again reworded for clarity, consistency, and grammatical inadequacies (see Additional file 1: Appendix A).

#### Content validity index and content clarity average of the student questionnaire

Out of 28 items, 2 items had a CVI less than .70 and were hence removed. Among the remaining 26 items, 3 items had a CVI between .75 and .79. Two items were retained after modification according to the expert feedback; however, 1 item was removed because of its similarity to another item. Twenty-three items had a CVI higher than .79. All items were retained except for 2 items that had a similar meaning as other items. Overall, 5 items were deleted. Version 3 of the questionnaire had 23 items with an SCVI of .94.

Regarding the content clarity, out of 23 items, 2 items had a CCA of 2, 18 had a CCA from 2.75 to 2.93 while three had a CCA from 2.46 to 2.68. The average clarity of the scale was 2.88 (see Additional file 1: Appendix B).

#### Response process validity of Teacher’s questionnaire through cognitive interviews

Table 3 shows that after establishing the content validity, 53 items remained in the questionnaire. Out of the 53 items, 42 items were found to be easily understood by the participants and required no change. Ten items needed more clarification and hence were explained in more detail by adding examples. One item was deleted as its content was also repeated in the subsequent items.

#### Response process validity of Student’s questionnaire through cognitive interviews

Twenty-three items were tested for response process validity. Sixteen required no change as they had no ambiguities, whereas 7 items were modified by adding examples to them.

#### Establishing the construct validity and reliability of the questionnaires

 The KMO and Bartlett’s test of sphericity for teacher and student questionnaires were .942 and .879, which indicated an adequate sample size for factor analysis. The reliability of the items before conducting CFA was found to be .941 and .870 for the teacher and student questionnaires, respectively, hence no items were removed [34]. A one-factor model was generated for both models, which was found not to have a good fit. Afterwards, 12- and 7-factor models, as hypothesized by the authors based on published literature [2, 3] and expert validation, were developed and analysed. These models were reduced to 11 and 6 factors after the deletion of items and the use of modification indices to achieve an acceptable model. Goodness of fit indices were established for these models, however factor correlations higher than 1 were found between the constructs. To correct this, closely related factors were combined. For example, ‘irrelevant curriculum content’ and ‘low-quality assessment’ had a high factor correlation (> 1). They were combined to form a new factor ‘Educational Program’. Tables 4 and 5 show the final teacher questionnaire with 25 items measuring 6 constructs, and the student questionnaire with 14 items measuring 3 constructs, along with the Cronbach’s alpha of the subscale and Cronbach’s alpha if deleted of the item. The CITC of items of ‘disciplinary culture’ was .25, and of ‘institutional culture’ were in the range of .22 to .29. The final versions of valid and reliable teacher and student questionnaires are given in Additional file 1: Appendix D and Appendix E that can be used for assessment of curriculum viability.

**Table 4:** Teacher questionnaire (final version) with Cronbach’s alpha if deleted

		CAID of the subscales	CAID of the questionnaire
**1-Educational Program (EP)**			
1	The contents I teach to my students are relevant to the intended learning outcomes of the curriculum (e.g., doctor as a professional, leader, communicator, researcher, etc.).	.59	.89
2	In my institution, the content taught in one course/module helps the students to understand the related concepts in other courses/modules.	.62	.89
3	The curricular content taught in my institution contributes to making students good doctors.	.64	.89
4	I use different assessment tools to assess knowledge, skills, and attitude in a course.	.62	.89
5	I construct assessment items according to the blueprinting for an exam.	.75	.90
6	I provide regular constructive feedback to my students.	.62	.89
*Cronbach alpha of the subscale*		*.69*	
**2-Disciplinary cultures (DC)**			
7	The attendance of faculty on campus is strictly monitored through biometric thumb impressions.	–	.89
8	Students are fined if they do not adhere to institution policies.	–	.89
*Cronbach alpha of the subscale*		*.41*	
**3-Social interaction (SI)**			
9	My institution offers formal opportunities for enhancing social interaction on educational issues among students.	.50	.89
10	My institution provides interactive online discussion forums.	.58	.89
11	My institution has meeting places for students and teachers for interaction.	.65	.89
*Cronbach alpha of the subscale*		*.67*	
**4-Institutional policies (IP)**			
12	Faculty can appeal against institutional decisions without any fear.	.70	.89
13	My institution’s decisions are based on defined policies and procedures.	.67	.89
14	I have been provided with a clear job description.	.70	.89
15	My institution gives awards for educational innovation (e.g., development of a new assessment tool, teaching method etc.).	.72	.89
16	My teaching and research activities are considered equally important for my promotion.	.75	.89
*Cronbach alpha of the subscale*		*.75*	
**5-Communication Practices (CP)**			
17	In my institution, there are no restrictions on the use of social media such as YouTube, WhatsApp etc. for educational purposes.	.74	.89
18	In my institution, regular faculty meetings are held at departmental level where everyone has the right to voice their concerns.	.68	.89
19	In my institution, the curriculum managers clearly communicate educational changes to the faculty.	.72	.89
20	In my institution, the faculty share strategies for effective classroom management among themselves.	.67	.89
21	In my institution, the faculty share their experiences of various instructional designs (e.g., 4C ID, Gagne 9 events) amongst them.	.69	.89
22	My institutional management shares the educational courses/modules in the curriculum with the faculty.	.68	.89
*Cronbach alpha of the subscale*		*.73*	
**6-Faculty involvement (FI)**			
23	I am invited to the meetings in which curricular issues are discussed and decisions are made.	.53	.89
24	My suggestions to update a course/module are given due consideration by committees that make curricular changes.	.46	.88
25	I have the authority to update the content of course/module in the curriculum.	.77	.89
*Cronbach alpha of the subscale*		*.68*	
*Overall internal consistency of the questionnaire*		*.89*

**Table 5:** Student questionnaire (final version) with Cronbach’s alpha if deleted

		CAID of the subscale	CAID of the questionnaire
**1-Educational Program (EP)**			
1	The contents taught to me are relevant to the intended learning outcomes of the curriculum (e.g., doctor as a professional, leader, communicator, researcher, etc.)	.68	.80
2	The curricular content taught in my institution contributes to making students good doctors.	.67	.80
3	In my institution, the content taught to me in one course/module helps me to understand the related concepts in other courses/modules.	.67	.80
4	I am assessed according to intended learning outcomes of the course.	.82	.83
5	My institution uses multiple assessment tools for the assessment of students.	.74	.80
*Cronbach alpha of the subscale*		*.76*	
**2-Student requirements (SR)**			
6	My institution offers appropriate Information Communications Technology facilities (e.g., the Internet, computers, software, etc.) for students.	.63	.81
7	My institution has an appropriate infrastructure that supports educational activities such as lectures, PBL sessions, skill acquisition, etc.	.60	.81
8	My institution has adequate support services such as counseling, scholarships, etc. for students.	.61	.81
9	In my institution, a student’s evaluation of the assessments/examinations is considered important for making changes in them.	.61	.81
10	In my institution, students are encouraged to ask questions during teaching sessions.	.68	.82
*Cronbach alpha of the subscale*		*.68*	
**3-Institutional Culture (IC)**			
11	Students are fined if they do not adhere to institution policies.	.42	.83
12	Student attendance is strictly monitored through biometric thumb impression in my institution.	.40	.83
13	My institution provides opportunities for social interaction between students and teachers.	.36	.81
14	My institution provides interactive online discussion groups	.36	.82
*Cronbach alpha of the subscale*		.46	
*Overall internal consistency (Cronbach’s alpha) of the questionnaire*		*.83*	

Table 6 shows the goodness-of-fit for these models, reported through ChiSq/df, RMSEA, CFI, NFI, TLI, GFI, and AGFI. Reliabilities of the teacher and student questionnaires were, in order, .901 and .834.

**Table 6:** Models and Confirmatory factor analysis indices

Model	ChiSq/df	GFI	RMSEA	TLI	CFI	AGFI	NFI
**Teacher questionnaire**							
1-factor model (52 items)	3.117	.724	.064	.719	.730	.702	.648
12-factor model (52 items)	2.421	.814	.052	.811	.828	.788	.741
11-factor model (29 items)	1.662	.936	.036	.945	.957	.913	.900
6-factor model* (25 items)	1.660	.940	.035	.950	.958	.924	.901
**Student questionnaire**							
1-factor model (23 items)	2.488	.821	.078	.766	.788	.785	.693
7-factor model (23 items)	1.874	.882	.060	.863	.887	.884	.790
6-factor model (17 items)	1.405	.937	.041	.957	.968	.905	.900
3-factor model* (14 items)	1.236	.953	.031	.974	.980	.931	.904

*without higher correlation factors > 1

**Fig. 2:**
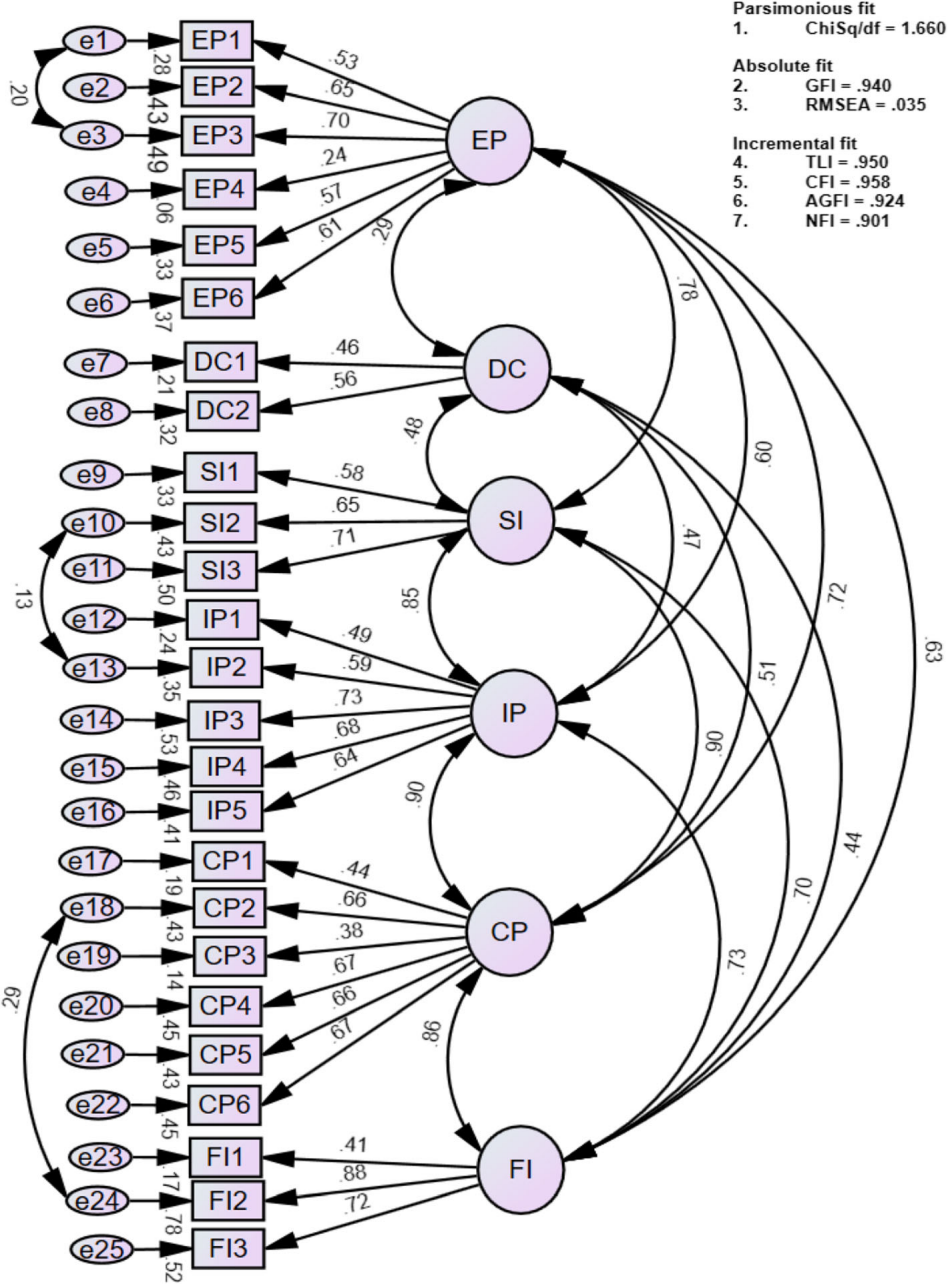
Sequential Equation Model for Teacher Questionnaire. The figure shows factor loadings, factor co-relations and good for fit indices (parsimonious, absolute, and incremental fit) for six factor model containing 25 items. Abbreviations used: EP = Educational Program, DC = Disciplinary Culture, SI = Social Interaction, IP = Institutional Policies, CP = Communication Practices, FI = Faculty Involvement, AGFI = Adjusted goodness of fit index, CFI = Comparative fit index, GFI = Goodness-of-fit index, NFI = Normed fit index, RMSEA = root mean square error of approximation, TLI = Tucker Lewis Index, χ^2^/df = Chi-square difference

**Fig. 3:**
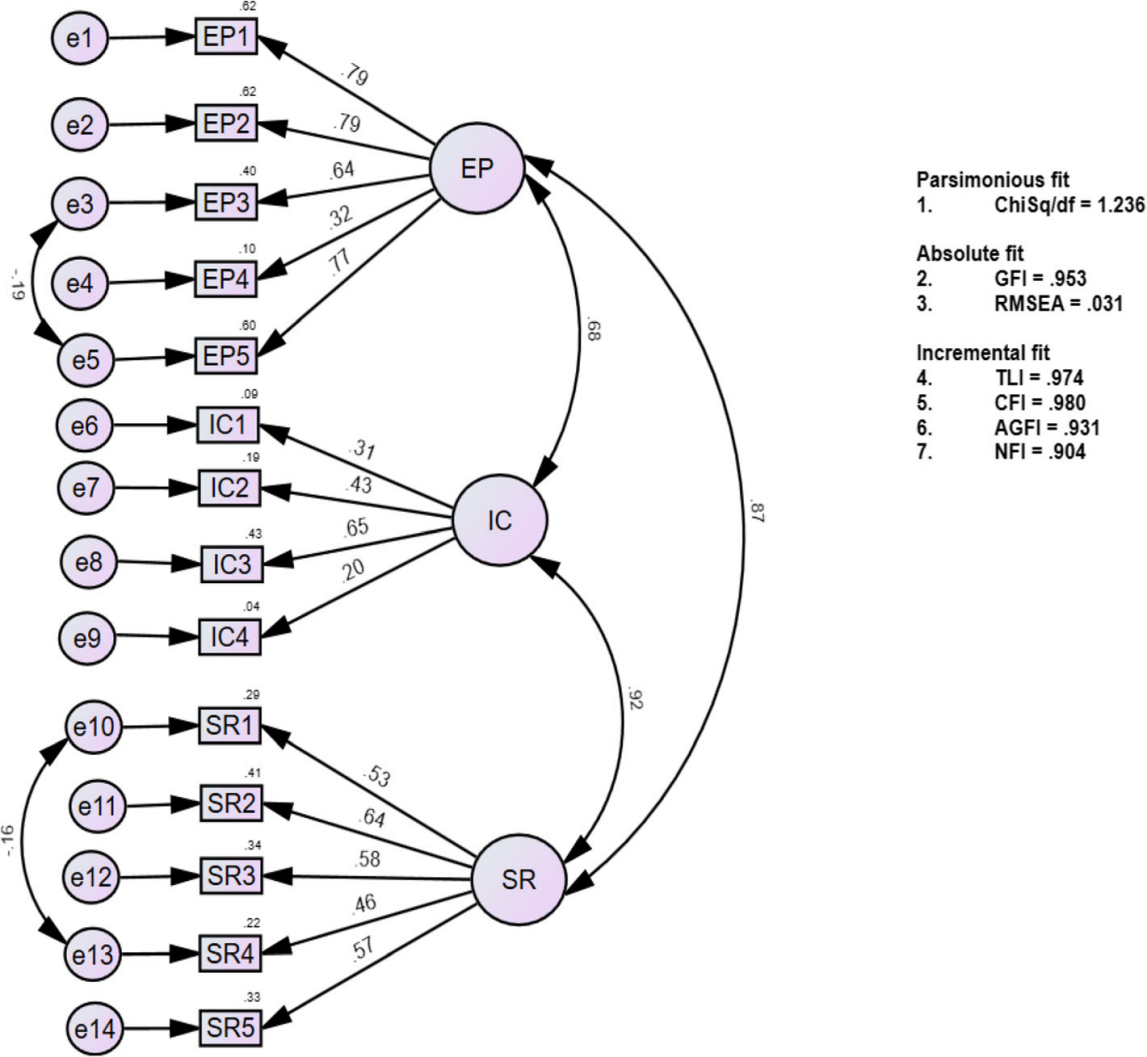
Sequential Equation Model for Student Questionnaire. The figure shows factor loadings, factor co relations and good for fit indices (parsimonious, absolute, and incremental fit) for three factor model containing 14 items. Abbreviations used: EP = Educational Program, IC = Institutional Culture, SR = Student Requirements

This represented parsimonious, absolute, and incremental fit for our models, shown through sequential equation models in Figs. 2 and 3, respectively. The figures show 6- and 3-factor models with 25 and 14 items, respectively, for the teacher and student questionnaires with all factor correlations being below 1.

## Discussion

The main objective of the study was to develop two valid and reliable questionnaires that can measure curriculum viability inhibitors, so that curriculum reviewers, developers, and implementers can use these questionnaires to identify the inhibitors in the implemented curriculum based on the feedback of faculty and students.

Many questionnaires that measure teacher and student perceptions about educational environments have been reported in the literature, [5, 7, 35, 36] but not on curriculum viability inhibitors explicitly. Through this study, we have developed two valid and reliable questionnaires that collectively identify curriculum viability inhibitors. The teacher questionnaire in our study covers the educational content and assessment, faculty involvement, institutional policies, social interaction, disciplinary culture, and communication practices. In comparison with the ‘Assessment of medical education environment by Teachers’(AMEET) questionnaire [4, 37], our questionnaire covers a wider range of areas of the curriculum. The AMEET addresses the educational environment in areas like perception of teaching, learning activities, students’ learning and collaborative atmosphere, and professional self-perception. Though it covers the educational environment in detail, it does not focus on social interaction, institutional policies, communication practices and faculty involvement relevant to the inhibitors of the curriculum. Regarding the student’s perception about the medical education curriculum, questionnaires that measure learning environments include the Health Professions Learning Environment Survey (HELES) [5], Johns Hopkins Learning Environment Scale (JHLES) [6], and Dundee Ready Educational Environment Measure (DREEM) [35]. These questionnaires focus on the learning environment of the institution. For instance, DREEM addresses the students’ perception of learning, teachers, atmosphere, and students’ academic self-perceptions and social self-perceptions. However, the student questionnaire in our study focuses specifically on the curriculum viability inhibitors that affects the curriculum such as irrelevant curriculum content and low-quality assessment. In addition, it also addresses issues such as student requirements, presence of strong disciplinary cultures and lack of social interaction. Also, student questionnaire in our study has two common constructs with the teacher questionnaire.

This study shows that teachers and students have their own perceptions of the same curriculum as reported by Konings etal [38]. Eight items under two constructs (Educational program and Institutional culture) related to learning outcomes, curricular content, assessment, disciplinary culture, and social interaction are identical in the teacher and student questionnaires developed in our study. Thus, these questionnaires will inform program evaluators about the congruence or disagreement between students and teachers in these areas. In case of congruence, responses will strengthen the diagnosis of curriculum inhibitors; however, a differing opinion will require further investigation, such as qualitative inquiry based on interviews or focus group discussions with the faculty on the areas where a differing opinion has been reported.

A main strength of our study was the extensive method of developing the questionnaires as per the guidelines and steps reported in the literature [9, 27, 29, 33, 39–41]. It also became clear that having two different questionnaires for students and teachers is necessary. Another strength of our study was that the teacher respondents in our study belonged to 77 medical colleges with varied experience, from junior to senior academic positions and involved in teaching different curriculum (Table 2).

Analysis of internal consistency using Cronbach’s α showed an acceptable level of internal consistency for the total scales (.89 and .83 for teacher and student questionnaires, respectively) and subscales (.67 to .76) identified from the confirmatory factor analysis (Figs. 2 and 3) for the ‘educational program’, ‘social interaction’, ‘institutional policies’, ‘communication practices’, ‘faculty involvement’ for the teacher questionnaire and ‘educational program’ and ‘student requirements’ for the student questionnaire. This is consistent with the alpha values reported in the literature [24, 42–44]. Two of the subscales ‘disciplinary culture’(2 items) in the teacher questionnaire and ‘institutional culture’ (4 items) in the student questionnaire had low internal consistency in the range of .41and .46, respectively. However, subscale with value less than .40 (Cronbach’s *α* = 0.37) has been retained in a questionnaire if it was unidimensional with fewer number of items [45], which was the case for the two subscales (Tables 4 & 5) in our study. Furthermore, values of Cronbach’s alpha less than 0.7 are common for one-dimensional scales with less than 10 items and have been justified in the literature [46–48]. In addition, regarding both these sub-scales in our study, they were an important measure of discipline and social activities regarding the institutional culture. Hence another reason to retain the items in these subscales was to maintain the content validity [46, 49]. Also the corrected item-to-total correlation (CITC) for all items in these subscales was > 0.2, which confirmed that each item belonged to its corresponding subscale [25, 26]. CITC is another measure of internal consistency and values between .2 to .4 are indicative that the items in the subscales are good measure of the corresponding construct [26, 50].

The study was not without limitations. We recruited participants in a ratio of 1:10 for the items in a questionnaire, which is considered adequate-to-good for the sample size. However, it is generally accepted that a larger sample size is better [17]. The sample size in ratios of 1:20 has been recommended [51]. Recruiting more participants may have yielded even better models. Another limitation of our study is that the confirmatory factor analysis was conducted in medical schools of mainly one country. However, teachers and students were from 77 and 12 medical colleges, respectively, experiencing different models of curricula. It is therefore expected that these questionnaires will be valid and reliable for different models of curriculum.

We advocate using these two questionnaires to identify issues in a curriculum that inhibit the achievement of quality standards. We further recommend that construct validity of the questionnaires be established in other countries, especially where the need for translation of the questionnaires will be required. To allow for difference in opinion of student and teachers about certain areas of the curriculum, we suggest further research to identify the reasons and their solutions for this difference in opinion, which can be a foundation for improving these questionnaires.

## Conclusion

We have developed valid and reliable teacher and student questionnaires that can be used to identify the inhibitors of curriculum viability. These questionnaires can be used by medical colleges to identify the inhibitors that hamper the achievement of quality standards. This will help in proposing solutions to address the inhibitors and improve the quality of the curriculum and will be preventive in nature to prepare for possible issues.

## Supplementary Information

**Additional file 1.** Appendix A. Teacher questionnaire and its modification based on content validity. Appendix B. Student Questionnaire and its modification based on content validity. Appendix C. Response process validity. Appendix E. Student questionnaire (Final version).

## Data Availability

The data generated and analysed during the study are available on request.

## Abbreviations

AIM: Assessment Implementation Measure
DREEM: Dundee Ready Educational Environment Measure
HELES: Health Education Learning Environment Measure
JHLES: Johns Hopkins Learning Environment Scale
PhD: Doctor of Philosophy
