# Supplementary material for: Development and validation of teacher and student questionnaires measuring inhibitors of curriculum viability
Source: BMC Med Educ. 2021 Jul 28;21:405. doi: 10.1186/s12909-021-02843-0 (PMC8317403; doi:10.1186/s12909-021-02843-0)
Supplement: Supplementary file 1 — Additional file 1. Appendix A. Teacher questionnaire and its modification based on content validity. Appendix B. Student Questionnaire and its modification based on content validity. Appendix C. Response process validity. Appendix E. Student questionnaire (Final version). [file 12909_2021_2843_MOESM1_ESM.docx]

**Appendix A: Teacher questionnaire and its modification based on content validity**

| **Constructs (Factor/Latent variables)** | **Items to measure curriculum inhibitors (Observed variables)** | **I-CVI** | **CCA** | **Decision** |
| --- | --- | --- | --- | --- |
| **Irrelevant curriculum content** | 1. Content of the course is according to the curricular learning outcomes. | 1 | 2.9375 | AM |
|  | 1. The topics I teach are relevant to the outcomes of the course. | 1 | 2.75 | AM |
|  | 1. The content helps the students to understand other related concepts. | 0.75 | 2.8125 | AM |
|  | 1. The curricular content contributes to making students better health professionals. | 0.875 | 2.6875 | AM |
| **Lack of resources in an institution** | 1. There are adequate resources available to me to facilitate my teaching activities. | 1 | 2.875 | AM |
|  | 1. The institution provides appropriate IT facilities to facilitate my teaching activities. | 1 | 2.9375 | AM |
|  | 1. The institution has the infrastructure that supports educational activities such as lectures, PBL sessions, skills acquisition, etc. | 1 | 2.875 | AM |
|  | 1. I have adequate access to library facilities and online resources. | 0.875 | 2.9375 | AM |
|  | 1. I have ample support staff to facilitate my teaching activities. | 0.75 | 2.75 | AM |
|  | 1. I have enough time to prepare for my teaching activities. | 0.875 | 3 | *A* |
| **Low-quality assessment** | 1. The assessment in my course is according to intended learning outcomes. | 1 | 2.75 | *A* |
|  | 1. I use different assessment tools to assess knowledge, skills, and attitude in a course effectively. | 1 | 2.75 | AM |
|  | 1. I conduct regular formative assessments for my students. | 1 | 2.9375 | *A* |
|  | 1. I provide regular constructive feedback to my students | 1 | 2.9375 | *A* |
|  | 1. I construct assessment items according to the table of specification for an exam | 1 | 2.875 | AM |
| **Lack of sufficient time for studying** | 1. My students have sufficient time for self-study in their schedule. | 1 | 2.9375 | *A* |
|  | 1. Students have adequate time off to study for exam preparation. | 1 | 2.9375 | AM |
|  | 1. The timetable is overloaded with planned teaching activities. | 0.875 | 2.5625 | AM |
| **Neglecting student needs and requirements** | 1. Students have significant participation in our educational committees. | 1 | 2.6875 | AM |
|  | 1. Students have an important input in our educational committees. | 0.8125 | 2.625 | **D** |
|  | 1. Student evaluations and feedback are considered important for making changes in the courses. | 1 | 2.625 | AM |
|  | 1. Student evaluations and feedback are considered for modifying assessments. | 1 | 2.6875 | AM |
|  | 1. Students input is taken in developing new courses according to their passion and need. | 0.6875 | 2.5625 | **D** |
|  | 1. I design my teaching activities to fully engage my students in teaching and learning activities. | 1 | 2.75 | AM |
|  | 1. I encourage my students to ask questions during my teaching activities. | 1 | 3 | *A* |
| **Presence of strong disciplinary cultures** | 1. There is a strong hierarchy of positions within the departments of my organisation. | 0.8125 | 2.6875 | AM |
|  | 1. The presence of faculty on campus is strictly monitored through biometric thumb/attendance. | 0.75 | 2.8125 | AM |
|  | 1. Students are fined if they do not adhere to institution policies. | 0.8125 | 2.9375 | A |
|  | 1. Student committees and societies are (strictly) monitored by the faculty/administration. | 0.6875 | 2.8125 | **D** |
| **Lack of social interaction** | 1. My institution offers formal opportunities for enhancing social interaction on educational issues among students. | 1 | 2.8125 | A |
|  | 1. Interactive online discussion sessions are provided by my institution. | 1 | 3 | AM |
|  | 1. My institution has meeting places for students and teachers for interaction. | 0.875 | 2.875 | A |
| **Research culture and patient care undervaluing education** | 1. My teaching activities are appreciated similar to research and/or clinical activities by the institution. | 0.8125 | 2.75 | AM |
|  | 1. The institution gives awards for educational innovation. | 1 | 2.8125 | AM |
|  | 1. Faculty gives more importance to research and/or patient care than teaching students. | 1 | 2.875 | AM |
|  | 1. The institution provides more resources for research than for teaching. | 0.9375 | 2.6875 | AM |
|  | 1. My teaching and research activities are considered equally important for my promotion. | 1 | 3 | A |
|  | 1. I have ample funding for patient care compared to funding for my teaching activities. | 0.625 | 2.625 | **D** |
| **Lack of policies and procedures** | 1. Educational committees in my institution consistently follow the laid-down procedures. | 1 | 2.9375 | AM |
|  | 1. My institution provides a clear educational vision and mission. | 1 | 2.9375 | A |
|  | 1. My institution has clear policies and procedures that support the teaching and learning process. | 1 | 3 | A |
|  | 1. I have been provided with a clear job description. | 1 | 3 | A |
|  | 1. Institution’s decisions are based on defined policies and procedures. | 1 | 3 | AM |
|  | 1. Faculty can appeal against organizational decisions without any fear. | 0.875 | 2.875 | AM |
|  | 1. Educational committees can rely on clearly formulated policies that help them to perform their tasks. | 1 | 2.9375 | **D** |
| **Leaders acting as Communication gatekeepers** | 1. There are no restrictions on the use of social media in the institution for educational purposes. | 1 | 2.9375 | AM |
|  | 1. We have regular faculty meetings held at the departmental level where everyone has the right to voice their concerns. | 1 | 2.9375 | AM |
|  | 1. Appropriate actions are taken on the concerns raised in the faculty meetings. | 1 | 3 | AM |
|  | 1. Educational changes are clearly communicated to the faculty. | 1 | 2.875 | AM |
| **Lack of staff involvement in organizational decision**  . | 1. I am invited to the meetings in which curricular issues are discussed and decisions are made. | 1 | 3 | A |
|  | 1. I am not encouraged to attend meetings in which curricular issues are discussed and decisions are made. | 0.8125 | 2.625 | **D** |
|  | 1. My suggestions are given due consideration by committees that make curricular changes. | 1 | 3 | A |
|  | 1. I have the authority to update the content of my courses. | 0.9375 | 2.8125 | AM |
|  | 1. Timetable is developed by keeping the commitments of the faculty involved. | 1 | 2.8125 | AM |
|  | 1. I can manage my workload as per my educational activities. | 0.9375 | 2.75 | A |
|  | 1. I attend regular meetings for teachers and students in our institution. | 1 | 2.8125 | **D** |
| **Lack of sharing best practices across organisation** | 1. Effective support for student learning is ensured through regular meetings of students and teachers. | 0.68 | 2.6875 | **D** |
|  | 1. We share strategies for effective classroom management among our colleagues. | 1 | 2.9375 | AM |
|  | 1. We share effectiveness of different instructional designs amongst the faculty. | 1 | 2.75 | AM |
|  | 1. Educational courses and programs are shared with the faculty. | 1 | 2.75 | AM |

(A=Accepted, AM=Accepted after modification, D=Deleted)

**Appendix B: Student Questionnaire and its modification based on content validity**

| **Domain**  **(Inhibitors)** | **Items to measure curriculum inhibitors** | **I-CVI** | **CCR** | **Decision** |
| --- | --- | --- | --- | --- |
| **Irrelevant curriculum content** | 1. Content of the courses taught to me is according to the stated learning outcomes. | 1 | 2.9375 | AM |
|  | 1. My teachers teach course contents aligned to the topic being taught. | 1 | 2.6875 | AM |
|  | 1. The content of the courses that I follow is irrelevant to my future professional requirements/job. | 0.75 | 2.6875 | AM |
|  | 1. The content of the courses that I follow helps me to understand other related topics. | 0.75 | 2.8125 | AM |
| **Lack of resources in an institution** | 1. My institution offers appropriate IT facilities. | 1 | 3 | AM |
|  | 1. The institution has an appropriate infrastructure that supports educational activities such as lectures, PBL sessions, skill acquisition, etc. | 1 | 2.8125 | AM |
|  | 1. Adequate library facilities are available in our institution. | 1 | 3 | AM |
|  | 1. Adequate student support services are available for my help | 0.8125 | 2.6875 | AM |
| **Low-quality assessment** | 1. I am assessed according to intended learning outcomes of the course. | 1 | 3 | A |
|  | 1. The institution uses multiple assessment tools for the assessment of students. | 1 | 2.9375 | AM |
|  | 1. Teachers provide regular feedback to students on educational activities to enhance their learning. | 1 | 2.9375 | AM |
|  | 1. Regular feedback is provided to us by our teachers. | 1 | 2.9375 | D |
| **Lack of sufficient time for studying** | 1. There is sufficient time allocated in the curriculum for self-study | 1 | 2.9375 | AM |
|  | 1. There is insufficient time for preparation for exams. | 1 | 2.9375 | AM |
|  | 1. I am happy with the preparatory leaves before exams. | 0.75 | 2.875 | D |
|  | 1. The timetable is overloaded with teaching and learning activities. | 0.8125 | 2.9375 | AM |
| **Neglecting student demands** | 1. Students have an important input in educational committees of the institution. | 1 | 2.9375 | AM |
|  | 1. Student evaluations and feedback are considered important for making changes in the courses. | 0.8125 | 2.75 | AM |
|  | 1. Student evaluations and feedback are considered for modifying assessments. | 0.8125 | 2.8125 | A |
|  | 1. Students input is taken in developing new courses according to their passion and need. | 0.625 | 2.5625 | D |
|  | 1. Students are encouraged to ask questions during teaching sessions. | 1 | 3 | AM |
| **Presence of strong disciplinary cultures** | 1. Students are fined if they do not adhere to institution policies. | 1 | 2.9375 | A |
|  | 1. Student committees are strictly monitored by the faculty. | 0.6875 | 2.8125 | D |
|  | 1. Student presence is strictly monitored through biometric thumb/attendance. | 0.875 | 2.8125 | AM |
| **Lack of social interaction** | 1. My institution provides opportunities for social interaction between students and teachers. | 1 | 2.9375 | A |
|  | 1. My institution provides formal opportunities for social interaction among students. | 1 | 3 | A |
|  | 1. My institution provides interactive online discussion forums | 1 | 2.9375 | A |
|  | 1. My institution has meeting places for students and teachers for interaction. | 0.875 | 2.8125 | D |

(A=Accepted, AM=Accepted after modification, D=Deleted)

**Appendix C : Response process validity**

**Participants.** Interviews were held with 6 teachers and 3 students. Double the number of teachers were questioned compared to number of students, because the teacher questionnaire had more than twice the number of items as the student questionnaire. The selection criteria for teachers were: (1) more than 10 years of teaching experience, (2) involvement in curriculum development and evaluation, and (3) representation of teachers from basic and clinical sciences. All 3 students were selected from the final-year undergraduate medical program in their clerkship rotations, as it was expected that they would have good knowledge of the whole curriculum of their school as compared to students from the earlier years.

**Materials.** In version 3, the teacher questionnaire had 53 items measuring 12 constructs and the student questionnaire had 23 items measuring 7 constructs. We used a combination of ‘think aloud’ and ‘verbal probing’ techniques [10].

***Think Aloud Technique*.** The participants were asked to read the item silently and think aloud what comes to their mind after reading the item [12]. This was explained to them with the help of an example. All participants were asked about the number of windows in their room and then say out loud what they think when they look at the windows. Then they were asked to read the first item silently and explain out loudly what they have understood from it. Further prompts, if required, were: ‘Please say out loud what are you thinking’ or ‘What goes through your mind?’ The participants were encouraged by praising them, so they continued to think aloud.

***Verbal Probing.*** In verbal probing, scripted or spontaneous questions were asked by the interviewer, after the participant read an item [13]. The scripted probes used were: ‘What does this term mean to you?’, ‘Can you explain it more’, ‘What do you think this item is inquiring?’, ‘Can you rephrase this item in your own words?’ and ‘What is the reason for selecting this response for this item?’ Examples of spontaneous probes that we used were: ‘Can you differentiate between intended learning outcomes and learning objectives?’, ‘What do you think the term better health professionals mean?’ and ‘How much time would be enough for preparing your teaching activity?’

We combined verbal probing with the think-aloud technique as ‘think aloud’ acts as a cue for respondents, to yield additional information on the quality of the items as explained in the procedure section below.

**Procedure**

***Test Interviews (Pilot).*** Test interviews were conducted with 1 co-author, 1 teacher, and 1 student using Zoom (zoom.us) to identify possible issues related to combining recorded think-alouds as cues for verbal probing. The time participants needed to answer the items in the questionnaire was also determined.

The average cognitive interview lasted approximately 60 minutes for 27 items in the teacher questionnaire and 50 minutes for 23 items in the student questionnaire. However, in verbal probing, after a few items (5-6), we observed that respondents had difficulty in remembering the think-aloud of the former items as compared to when they were asked a probing question directly after each item. Regarding cued retrospective probing [14], the primary researcher played the recorded think-aloud back to the participant and explored the items with scripted and spontaneous probes. We found that it yielded no extra benefit in providing a cue as compared to the combination technique of verbal probing after each think-aloud item. It was also observed that it required more time and was less feasible as the recording had to be played back to the interviewee on Zoom.

***Cognitive Interviews for the study***. The cognitive interview requires a sustained concentration on behalf of the participants [15]. Hence protocols regarding these interviews were planned based on the pilot interviews. For the teacher questionnaire, we divided the 53 items in the questionnaire between 2 participants, so that participants 1 and 2 had to address 27 and 26 items each, respectively. Therefore, 6 teachers responded to the teacher’s questionnaire, and 3 students responded to the student questionnaire, as it had only 23 items. To increase the credibility of the interview technique and reduce the primary researcher's (RAK) bias, another researcher (UM) was also present during each interview. The participants read the item silently. After reading each item silently, participants were requested to think aloud to express their understanding of the item. Based on the think-aloud and scripted probes, additional questions were asked to clarify the responses.

**Data Analysis.** Analytic memos (reflections of the researcher on the data) were created based on the think-aloud and verbal responses. These memos were coded into the following categories: (1) items with no problems in understanding, (2) items with minor problems in understanding, and (3) items with major problems in understanding [16]. These categories were assigned independently by RAK and UM. Items that required more clarity were reworded and further refined through review from the remaining co-authors (AS, MAL and JVM).

| **1-Educational Program (EP)** | | **Rating** | | | | |
| --- | --- | --- | --- | --- | --- | --- |
| 1 | The contents I teach to my students are relevant to the intended learning outcomes of the curriculum (e.g., doctor as a professional, leader, communicator, researcher, etc.). | 1 | 2 | 3 | 4 | 5 |
| 2 | In my institution, the content taught in one course/module helps the students to understand the related concepts in other courses/modules. | 1 | 2 | 3 | 4 | 5 |
| 3 | The curricular content taught in my institution contributes to making students good doctors. | 1 | 2 | 3 | 4 | 5 |
| 4 | I use different assessment tools to assess knowledge, skills, and attitude in a course. | 1 | 2 | 3 | 4 | 5 |
| 5 | I construct assessment items according to the blueprinting for an exam. | 1 | 2 | 3 | 4 | 5 |
| 6 | I provide regular constructive feedback to my students. | 1 | 2 | 3 | 4 | 5 |
| **2-Disciplinary cultures (DC)** | | | | | | |
| 7 | The attendance of faculty on campus is strictly monitored through biometric thumb impressions. | 1 | 2 | 3 | 4 | 5 |
| 8 | Students are fined if they do not adhere to institution policies. | 1 | 2 | 3 | 4 | 5 |
| **3-Social interaction (SI)** | | | | | | |
| 9 | My institution offers formal opportunities for enhancing social interaction on educational issues among students. | 1 | 2 | 3 | 4 | 5 |
| 10 | My institution provides interactive online discussion forums. | 1 | 2 | 3 | 4 | 5 |
| 11 | My institution has meeting places for students and teachers for interaction. | 1 | 2 | 3 | 4 | 5 |
| **4-Institutional policies (IP)** | | | | | | |
| 12 | Faculty can appeal against institutional decisions without any fear. | 1 | 2 | 3 | 4 | 5 |
| 13 | My institution’s decisions are based on defined policies and procedures. | 1 | 2 | 3 | 4 | 5 |
| 14 | I have been provided with a clear job description. | 1 | 2 | 3 | 4 | 5 |
| 15 | My institution gives awards for educational innovation (e.g., development of a new assessment tool, teaching method etc.). | 1 | 2 | 3 | 4 | 5 |
| 16 | My teaching and research activities are considered equally important for my promotion. | 1 | 2 | 3 | 4 | 5 |
| **5-Communication Practices (CP)** | | | | | | |
| 17 | In my institution, there are no restrictions on the use of social media such as YouTube, WhatsApp etc. for educational purposes. | 1 | 2 | 3 | 4 | 5 |
| 18 | In my institution, regular faculty meetings are held at departmental level where everyone has the right to voice their concerns. | 1 | 2 | 3 | 4 | 5 |
| 19 | In my institution, the curriculum managers clearly communicate educational changes to the faculty. | 1 | 2 | 3 | 4 | 5 |
| 20 | In my institution, the faculty share strategies for effective classroom management among themselves. | 1 | 2 | 3 | 4 | 5 |
| 21 | In my institution, the faculty share their experiences of various instructional designs (e.g., 4C ID, Gagne 9 events) amongst them. | 1 | 2 | 3 | 4 | 5 |
| 22 | My institutional management shares the educational courses/modules in the curriculum with the faculty. | 1 | 2 | 3 | 4 | 5 |
| **6-Faculty involvement (FI)** | | | | | | |
| 23 | I am invited to the meetings in which curricular issues are discussed and decisions are made. | 1 | 2 | 3 | 4 | 5 |
| 24 | My suggestions to update a course/module are given due consideration by committees that make curricular changes. | 1 | 2 | 3 | 4 | 5 |
| 25 | I have the authority to update the content of course/module in the curriculum. | 1 | 2 | 3 | 4 | 5 |

1 = strongly disagree, 2 = somewhat disagree, 3 = neither agree nor disagree, 4 = somewhat agree, and 5 = strongly agree

**Appendix E: Student questionnaire (Final version)**

| **1-Educational Program (EP)** | | **Rating** | | | | |  |
| --- | --- | --- | --- | --- | --- | --- | --- |
| 1 | The contents taught to me are relevant to the intended learning outcomes of the curriculum (e.g., doctor as a professional, leader, communicator, researcher, etc.) | 1 | 2 | 3 | 4 | 5 | |
| 2 | The curricular content taught in my institution contributes to making students good doctors. | 1 | 2 | 3 | 4 | 5 | |
| 3 | In my institution, the content taught to me in one course/module helps me to understand the related concepts in other courses/modules. | 1 | 2 | 3 | 4 | 5 | |
| 4 | I am assessed according to intended learning outcomes of the course. | 1 | 2 | 3 | 4 | 5 | |
| 5 | My institution uses multiple assessment tools for the assessment of students. | 1 | 2 | 3 | 4 | 5 | |
| **2-Student requirements (SR)** | | | | | | |  |
| 6 | My institution offers appropriate Information Communications Technology facilities (e.g., the Internet, computers, software, etc.) for students. | 1 | 2 | 3 | 4 | 5 | |
| 7 | My institution has an appropriate infrastructure that supports educational activities such as lectures, PBL sessions, skill acquisition, etc. | 1 | 2 | 3 | 4 | 5 | |
| 8 | My institution has adequate support services such as counseling, scholarships, etc. for students. | 1 | 2 | 3 | 4 | 5 | |
| 9 | In my institution, a student’s evaluation of the assessments/examinations is considered important for making changes in them. | 1 | 2 | 3 | 4 | 5 | |
| 10 | In my institution, students are encouraged to ask questions during teaching sessions. | 1 | 2 | 3 | 4 | 5 | |
| **3-Institutional Culture (IC)** | | | | | | |  |
| 11 | Students are fined if they do not adhere to institution policies. | 1 | 2 | 3 | 4 | 5 | |
| 12 | Student attendance is strictly monitored through biometric thumb impression in my institution. | 1 | 2 | 3 | 4 | 5 | |
| 13 | My institution provides opportunities for social interaction between students and teachers. | 1 | 2 | 3 | 4 | 5 | |
| 14 | My institution provides interactive online discussion groups | 1 | 2 | 3 | 4 | 5 | |

1 = strongly disagree, 2 = somewhat disagree, 3 = neither agree nor disagree, 4 = somewhat agree, and 5 = strongly agree
